# Concurrent validity and between-device reliability of the Catapult Vector S8 GNSS device

**DOI:** 10.1371/journal.pone.0333792

**Published:** 2025-10-30

**Authors:** Susanne Ellens, Codey Moran, Matthew C. Varley

**Affiliations:** 1 Sport, Performance, and Nutrition Research Group, School of Allied Health, Human Services and Sport, La Trobe University, Melbourne, Australia; 2 Catapult, Melbourne, Australia; Afyon Kocatepe University, TÜRKIYE

## Abstract

This study assessed the concurrent validity and between-device reliability of the Catapult Vector S8 GNSS device for measuring distance, speed, acceleration and banded distance metrics. Twelve male sub-elite team sport athletes completed a testing protocol consisting of linear sprints, change of direction drills and a modified small-sided game. Validity was evaluated against criterion reference systems, a VICON motion capture system for most trials and a Stalker ATS radar for 50 m sprints, evaluated using root mean square error (RMSE) and mean bias. Between-device reliability was assessed using interclass correlation coefficients (ICC) and typical error (TE%). The Vector S8 demonstrated good validity with minimal errors for instantaneous distance (RMSE: 0.03 ± 0.01 m), speed (RMSE: 0.14 ± 0.05 m·s^−1^) and acceleration (RMSE: 0.29 ± 0.14 m·s^−2^). No overall bias was detected for instantaneous distance and speed, and the bias for acceleration (−0.016) was minimal. Accumulated distance showed a small underestimation across trials (mean bias −1.42%), with consistently low RMSE values (0.26–3.06 m), indicating high measurement precision. The 50 m sprint results showed similar validity, with minimal RMSE for instantaneous speed (0.14 ± 0.15 m·s^−1^) and acceleration (0.22 ± 0.22 m·s^−2^). Between device reliability demonstrated excellent agreement across all measured variables (ICC ≥ 0.95) with good precision (TE as CV < 3.13%). No significant systematic bias was observed between devices for any variable (p > 0.05). This is the first study to validate the Catapult Vector S8 GNSS device, demonstrating that it is valid and reliable for measuring distance, speed and acceleration during sport-specific movements.

## Introduction

Global Navigation Satellite Systems (GNSS) are currently one of the most used technologies to track athletes [1–3]. The primary purpose of a GNSS is to quantify the external load of an athlete during training and matches [4,5]. External load is determined by collecting kinematic data such as distance covered, sprints, accelerations and distance covered at certain speeds [4]. Practitioners and researchers use this information to enhance performance, support recovery, and reduce injury risks [4,5]. To allow meaningful interpretations of the GNSS data, it is important that the GNSS devices are both valid and reliable, to allow the user to make well-informed decisions.

The validity of a GNSS device is defined as the degree to which the device accurately measures what it is intended to measure [6]. This is typically evaluated by comparing the GNSS data to a criterion measure (gold standard). It is important that each measure of a GNSS device, i.e., distance, speed, and acceleration, is assessed in a validation study. A crucial aspect of validity assessment is the selection of an appropriate criterion reference measure, where measuring tapes and timing gates are the most commonly used criterion reference measures [7]. A limitation of measuring tapes is that they do not capture the exact path travelled, especially by change-of-direction movements, potentially leading to inaccurate results. Furthermore, timing gates only provide average speeds over predefined distances (i.e., between two gates). Radar technology provides an alternative for instantaneous linear speed measurement and is considered a gold standard for determining peak speed [8,9]. However, radar technology cannot be used during sport-specific movements and is restricted to linear movements only. A 3D motion analysis system is considered a gold standard criterion for measuring instantaneous dynamic sport-specific movements and can be used during drills representing game-like situations [7]. The reliability of a GNSS device refers to the reproducibility of measures on repeat occasions [10]. When measurements of numerous GNSS devices are compared, which for example happens when comparing data of a squad of players, between-device reliability is important [8,11,12]. Between-device reliability is defined as the consistency of measurements between different devices when measuring the same movement [13].

Several factors influence the validity and reliability of GNSS devices. The sampling frequency of the GNSS device is a crucial factor affecting the validity and reliability, where higher sampling frequencies (≥ 10 Hz) have been reported to be more accurate compared to GNSS devices with a lower sampling frequency [8,14]. The number of satellites and the positioning of the satellites interacting with the GNSS devices influence accuracy [12]. The quality of the positioning of the satellites connected to the GNSS devices is defined by the horizontal dilution of precision (HDOP) [15]. A HDOP value of ≤ 1 represents the ideal positioning of satellites in the sky and results in greater accuracy; this also requires a low variability and high mean number (≥ 12) of satellites [15]. Multi-GNSS devices can connect to multiple satellite constellations (e.g., global positioning system (GPS), Galileo, GLONASS) concurrently, and therefore increase the number of satellites it can connect to. An increase in error of distance and speed measures have been linked to a decrease in connected satellites and increase in HDOP values [15–17].

Furthermore, changes in speed can compromise the validity of GNSS devices with a sampling frequency < 10 Hz, where low accuracy is shown for movements with a high change in speed and frequent changes of direction [9,18–20]. These GNSS devices with a sampling frequency < 10 Hz utilise one satellite constellation (GPS), which also adds to the cause of decreased accuracy of the devices [15]. However, GNSS devices with a sampling frequency ≥ 10 Hz and connectivity to multiple satellite constellations, are less heavily influenced and have smaller error values for distance-based measures for high changes in speed or frequent changes of direction movements [1,8,21,22]. However, the error in speed measures increases as the speed increases, where acceleration follows a similar pattern [21]. Research has also suggested that speed measured at lower speeds and likely higher accelerations are less accurate and slightly overestimated [1]. From a practitioner’s point of view, these errors can cause inaccuracies in commonly used acceleration variables such as high acceleration/deceleration efforts (± 3 m·s^−2^) and lower speed bands (e.g., 0–5 m·s^−1^) where higher accelerations can occur. Large differences in accuracy are present between GNSS device brands and models [8,14]. Therefore, validity and reliability studies should be conducted when new GNSS devices or models are released.

Catapult is one of the most widely used GNSS device brands in high-performance sport and research [1–3], making its validity and reliability important for practitioners and researchers. However, the validity and reliability of the Catapult S8 GNSS device have not yet been examined in the literature. Therefore, the aims of this study were to (I) investigate the concurrent validity of distance, speed, and acceleration data and (II) between-device reliability of the Catapult Vector S8 GNSS device. Validity was established by comparing the measures to a criterion reference system during small-sided games, linear and change-of-direction drills.

## Methods

### Participants

Twelve male sub-elite soccer and Australian football players (mean ± SD; age: 21.4 ± 2.6 years, height: 184.4 ± 7.5 cm, body mass: 83.7 ± 13.8 kg) volunteered to participate in this study. Participants were recruited for this study in the period from 23-Sep-2024 to 21-Oct-2024. Prior to participation, all participants were informed about the risks and benefits of the research before signing informed consent. The participants wore their normal training clothes during data collection, which took place over two days (one day apart). On each day, six players participated. All sessions took place after sunset using floodlights on an empty and open (not surrounded by stands or buildings) grass soccer field. The weather was dry and windless with temperatures of 14° ± 1° Celsius. All procedures of this study were approved by the Human Research Ethics Committee of La Trobe University (reference number: HEC24295).

A sample size estimation based on Bland-Altman plot analysis was used, which calculates the required sample size for a method comparison study. This was calculated using an expected mean difference of speed of 0.28 m·s^−1^ with a standard deviation of 0.07 m·s^−1^ [21]. The maximum allowed difference between methods used was 0.54 m·s^−1^ which was based on the industry standards set by the FIFA quality programme for electronic performance and tracking systems [23]. An α-level of 0.05 and β-level of 0.10 (power was 90%) were used. As this study investigated the validity and reliability of a GNSS athlete tracking device, the sample size is the total number of trials for each movement trial rather than the number of participants. As each participant performed each movement trial twice, the total number of participants needed was eight to get at least sixteen trials for each movement. To account for data loss and device faults, twelve participants were included. The total sample size for each movement trial in this study was above sixteen, the exact number of trials is provided in the *Movement trials* section of this manuscript.

### Validated system

Catapult Vector S8 10 Hz GNSS devices (Firmware: 0.3.0; Catapult Sports, Melbourne, Australia) were used for this study. The GNSS devices provided position (longitude and latitude), accumulated distance (m), instantaneous speed (m·s ⁻ ^1^) and acceleration (m·s ⁻ ^2^) data which were processed and downloaded using the manufacturer’s software (OpenField, version 3.13.0, Catapult Sports, Melbourne, Australia). The manufacturer’s software processed data were used for this study, as this is the data used by practitioners. Distance was determined based on positional differencing and the speed was Doppler-derived speed, which refers to speed measured by the shift in satellite signal frequency resulting from the movement of the GNSS device [24]. The acceleration data is subsequently derived from the Doppler-derived speed. Eleven different GNSS devices were used for this study. The GNSS devices were positioned in the manufacturer’s vest and placed between the participants’ shoulder blades. All GNSS devices were turned on 15 min prior to data collection to ensure each unit had a satellite lock. The study sample had an average ± SD horizontal dilution of precision (HDOP) of 0.99 ± 0.06 and 57.3 ± 1.78 number of satellites.

### Reference systems

**Fig 1:**
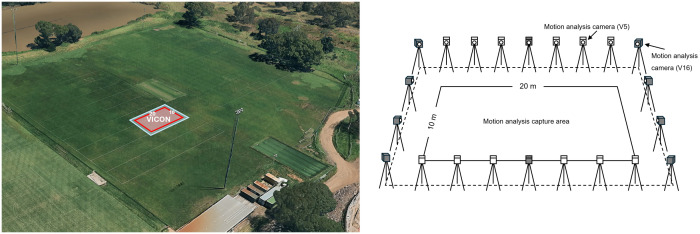
Motion analysis camera setup. Data collection test location (VICON) on the pitch (left). Schematic illustration of the motion analysis camera setup around the capture area where all trials were performed (right). The grey-filled motion analysis cameras refer to the Vantage 16 (V16) model, while the white-filled cameras represent the Vantage 5 (V5) model.

**Fig 2:**
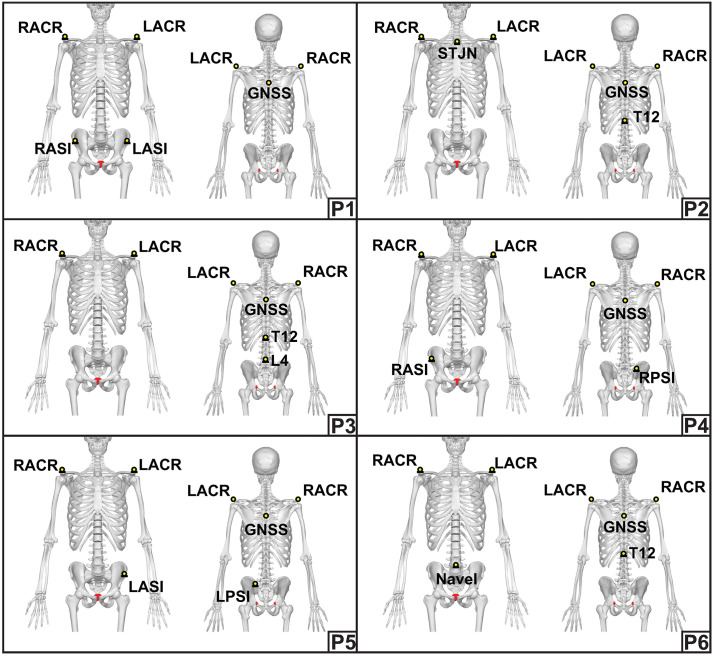
Participant markup. Unique markup and positions of the reflective markers for each of the six participants (P1-P6) in a data collection session.

A 3D infrared camera-based motion analysis system (VICON, Oxford, UK) capturing data at 100 Hz was used to determine the criterion reference for distance (m), speed (m·s ⁻ ^1^), and acceleration (m·s^−2^) data. The setup consisted of twenty-two cameras (ten Vantage 16 cameras, twelve Vantage 5 cameras, Software: Nexus 2.16) which were evenly spread around a 10 x 20 m capture area (Fig 1). Reflective markers with a diameter of 40.0 mm were used to ensure stable marker recognition within the capture area (10 x 20 m). Each participant in a session had a unique markup consisting of five markers, as can be seen in Fig 2. Only the marker corresponding to the location of the GNSS device was used for analysis. All other markers contributed to the unique markup and assisted in differentiating participants when multiple individuals were present in the capture area at the same time. Each marker was positioned on the participant’s skin using double sided tape and adhesive surgical tape on one of the following locations: middle of the GNSS device on the outside of the manufacturer’s vest (GNSS), right acromion (RACR), left acromion (LACR), right anterior superior iliac spine (RASI), left anterior superior iliac spine (LASI), right posterior superior iliac spine (RPSI), left posterior superior iliac spine (LPSI), sternum jugular notch (STJN), thoracic spine vertebra 12 (T12), lumbar spine vertebra 4 (L4), 1 cm below/inferior to the navel (NAVEL). The raw motion analysis data were filtered using a zero-lag fourth order low-pass Butterworth filter with a 3 Hz cut-off frequency which was determined based on residual analysis and choosing an equal balance between signal distortion and the amount of noise allowed through [21,25]. Gaps in the data ≤ 5 samples (0.05 s) were filled using spline interpolation. Gaps that were ≥ 5 samples (0.05 s) were excluded from analysis. The X and Y coordinate data corresponding to the middle of the GNSS device were exported and used for analysis. The z-coordinates (vertical axis) were excluded from analysis, because the S8 GNSS device does not collect data along the vertical axis.

Criterion measure of maximal speed, captured over 50 meters, was measured using a Doppler radar (Stalker ATS II, Plano, TX, USA, 47 Hz), as the motion capture area was not large enough to permit a 50 m sprint. Raw, unprocessed radar speed data were exported via proprietary software (Stalker ATS 5.1.1). The radar has a reported ± 0.045 m·s^−1^ accuracy within a 5-degree field from the radar [26].

### Movement trials

#### Validity.

**Fig 3:**
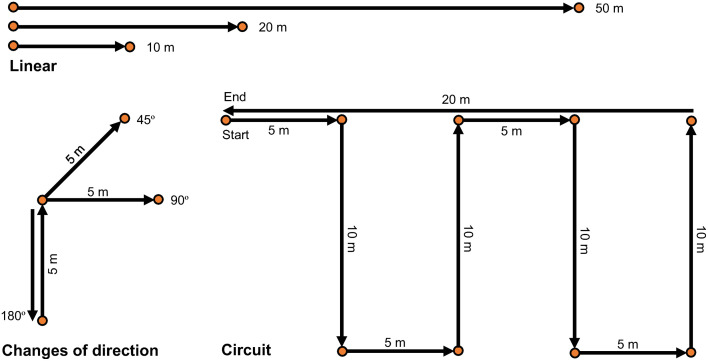
Illustration of movement trials. Linear, changes of direction and circuit exercises performed during the validation testing.

Seven different movement trials, illustrated in Fig 3, were performed by the participants with 60 s of passive recovery between trials. The following movement trials were included in the data collection: Change-of-direction trials of 45°, 90° and 180° consisted of a 5 m sprint into the change of direction followed by a 5 m sprint; Linear runs of 10 m, 20 m and 50 m; Circuit with a flying start consisting of sharp turns, accelerations and decelerations, totalling 70 m; Modified small-sided game (SSG) of 2 min, 3vs3 where it was the objective to pass a tennis ball among teammates and score points. Players needed to complete at least five successful passes before attempting to score by catching the ball in a 1 x 1 m goal area at their opponent’s end. Running with the ball was not allowed, forcing the players to create space by accelerating, decelerating, and performing changes of direction at a high intensity. It was instructed and encouraged during the SSGs that players without the ball keep moving at a high intensity. All participants started and ended each trial in a stationary position for 5 s and were instructed to perform to their maximal ability, with each trial performed twice. The different movement trials were identified in the data using the static standing periods. These periods were used as markers to segment the trials: the start was identified by an increase above zero in the speed data, while the end of each trial was identified by the speed returning to zero.

The total number of exercises included for analysis was 24 COD 45°, 24 COD 90°, 23 COD 180°, 24 linear 10 m, 24 linear 20 m, 17 linear 50 m, 18 circuit, 16 SSG. A total of 21, 50 m sprints were conducted, three participants performed the 50 m once. Four 50 m trials were excluded due to measurement errors from the radar device. For the motion analysis data, 14 data files (6 circuit and 8 SSG) contained data gaps and were excluded from analysis.

#### Between-device reliability.

**Fig 4:**
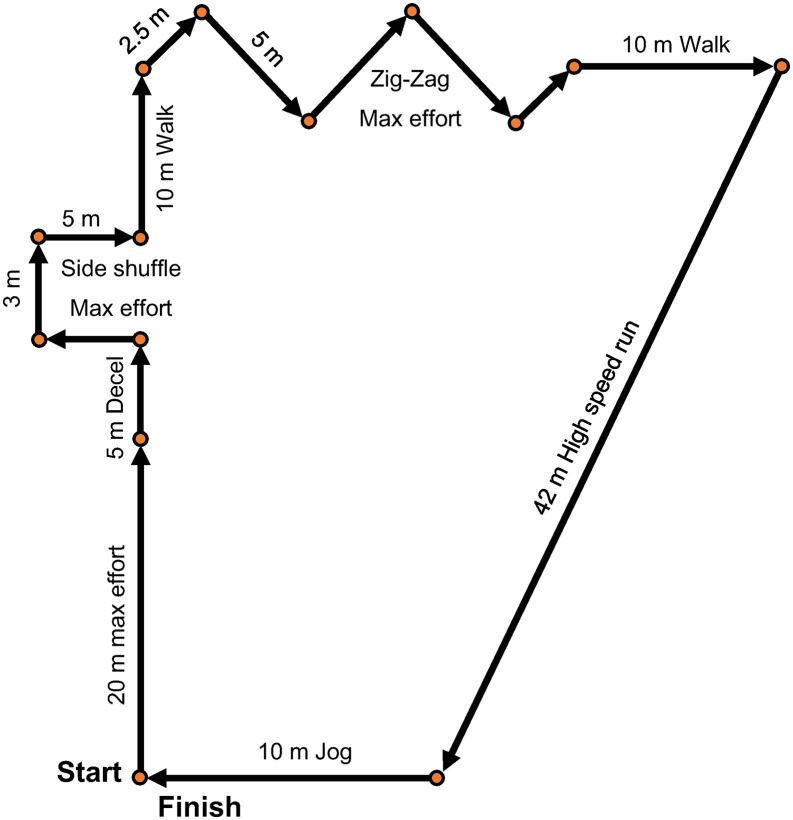
Between-device reliability protocol. Standardised intermittent running protocol for the between-device reliability trials. Decel = deceleration, High-speed run > 5 m·s^−1^.

Participants completed a standardised intermittent running protocol of ~139m while wearing two GNSS devices positioned between the scapulae in a custom multi-pouch garment. Each participant performed three trials of the protocol, with 60 s of passive recovery between trials, resulting in 36 trials included for analysis. The protocol included high-speed running (> 5 m·s^−1^), maximal efforts, jogging, walking, and changes of direction, as shown in Fig 4.

### Data analysis

The instantaneous distance (distance per sample) of the GNSS data was calculated by differencing the accumulated distance between two consecutive samples. The instantaneous distance of the motion analysis data was calculated by the Euclidean distance between two consecutive x and y coordinates. The speed of the motion analysis data was calculated by differentiating the positional data and applying the same filter as used in the manufacturer’s software (OpenField, version 3.13.0) on the GNSS data. Previous research has shown that different filters applied to the GNSS data can heavily influence and affect data [27–31]. It was therefore chosen to use the same filtering practices on the criterion data as those used on the GNSS data. This ensured that any differences present in the data were not due to the filtering practices used, but due to differences in the collected raw data. The filtering information was provided to the researchers by the manufacturer; however, details are not included here due to the manufacturer’s intellectual property. Similarly, acceleration was calculated by differencing the speed data and filtered using the manufacturer’s specifications. The derived motion analysis data were then synchronised to the GNSS data by cross-correlating the speed signals of each trial to find the time offset that maximised the correlation [32]. Cross correlation is a commonly used synchronisation approach in validation research [33–35], which can be further refined by assessing the similarity of the two datasets at a variety of time shifts using root mean square error (RMSE) [1,36]. The data of each trial were therefore further synchronised by shifting the motion analysis speed signal forwards and backwards by 50 data points in one-step increments, while keeping the GNSS speed signal fixed. The RMSE was established between both datasets (GNSS and motion analysis data) at each shift. The shift with the lowest RMSE was used to synchronise the datasets. The synchronised motion analysis data were down-sampled to 10 Hz to match the GNSS data and were used for analysis.

Raw, unprocessed radar speed was filtered using the same method as the manufacturer software. Acceleration was derived by differentiating the filtered speed, which was also processed based on the manufacturer’s specifications. The filtered radar data were down-sampled to 10 Hz and synchronized with the Catapult data by cross correlating the speed signals to determine the time offset that maximized the correlation. All data processing and analysis were conducted using the R statistical programming language (version 4.2.0) and the gsignal package.

### Statistical analysis

#### Validity.

The magnitude of the error and the relationship between the GNSS data and the criterion for distance, speed and acceleration were assessed using root mean square error (RMSE), mean bias and mean bias percentage error (%) [12]. The mean bias percentage error is defined as the percentage difference in relation to the criterion reference measure, calculated for each trial and averaged across each respective movement trial category. To quantify the difference between the criterion and GNSS for instantaneous distance, speed and acceleration, RMSE and mean bias were calculated for each trial by evaluating the instantaneous error (GNSS – Criterion) at each data point across the synchronized 10 Hz time series and presented as RMSE (mean ± standard deviation) and mean bias ± 90% confidence interval (CI).

#### Between-device reliability.

The between-device reliability of the Vector S8 device for measuring distance, speed and, acceleration was assessed using the following metrics: Total distance (m), maximal speed (m·s^−1^), banded distance (m) for low-speed running (LSR = 0–5 m·s^−1^) and high-speed running (HSR > 5 m·s^−1^), maximal acceleration (m·s^−2^), maximal deceleration (m·s^−2^), acceleration load, defined as the accumulated absolute acceleration values and high metabolic load distance (HMLD) defined as the distance covered above or equal to a metabolic power of 25.5 W·kg^−1^. The reliability was evaluated using typical error (TE) expressed as a coefficient of variation (CV, 90% CI) and intraclass correlation coefficients (ICCs, 90% CI) using a two-way mixed-effects model, absolute agreement, single measurement [37]. Reliability was classified as good if CVs were < 5%. ICCs were interpreted as follows: poor (< 0.5), moderate (0.5–0.75), good (0.75–0.9), and excellent (> 0.9) [38].

Systematic bias between devices was assessed using paired t-tests, with statistical significance set at p < 0.05. The effect magnitude was expressed in Cohen’s d standardised units using the following descriptors: trivial (< 0.2), small (0.2–0.5), medium (0.5–0.8), large (> 0.8). Normality was tested using the Shapiro-Wilk test. If the assumption of normality was violated, comparisons were made using the non-parametric Wilcoxon signed-rank test. Agreement and variability between devices were further examined using Bland-Altman analysis, which provided mean bias and 90% limits of agreement.

## Results

### Validity

**Fig 5:**
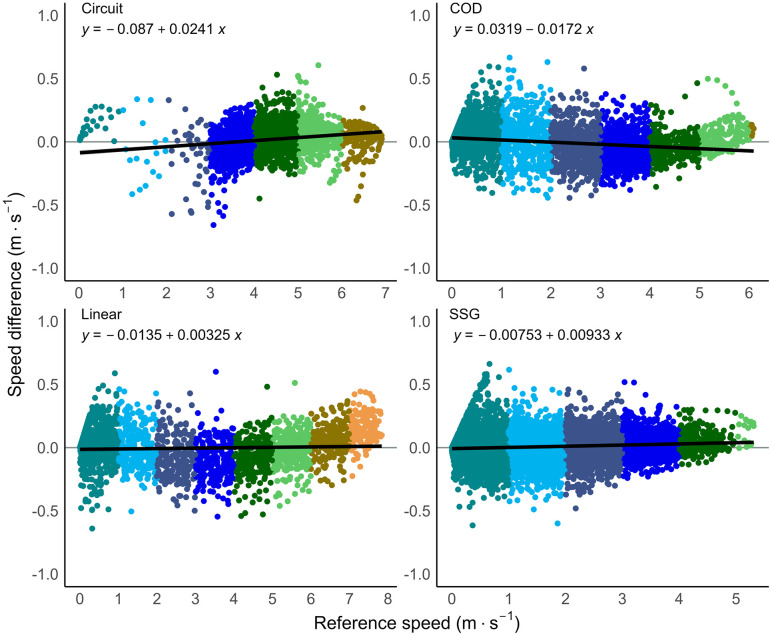
Difference in speed by movement category. Instantaneous criterion reference speed (m·s^−1^) of the motion analysis (MA) data compared to the global navigation satellite system (GNSS) data, divided into speed thresholds and split by movement category. Speed difference = MA – GNSS data. COD = change of direction trials, SSG = modified small-sided game trials.

**Fig 6:**
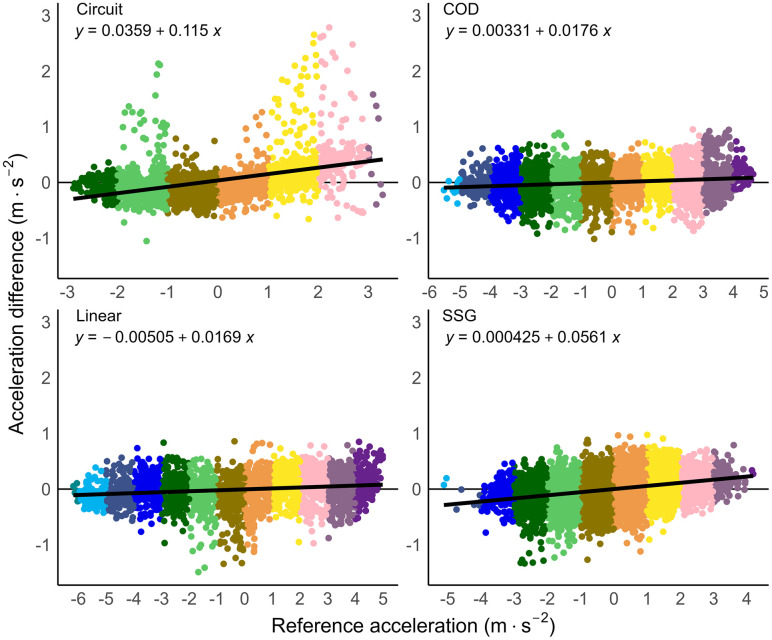
Difference in acceleration by movement category. Instantaneous criterion reference acceleration (m·s^−2^) of the motion analysis (MA) data compared to the global navigation satellite system (GNSS) data, divided into acceleration thresholds and split by movement category. Acceleration difference = MA – GNSS data. COD = change-of-direction trials, SSG = modified small-sided game trials.

**Fig 7:**
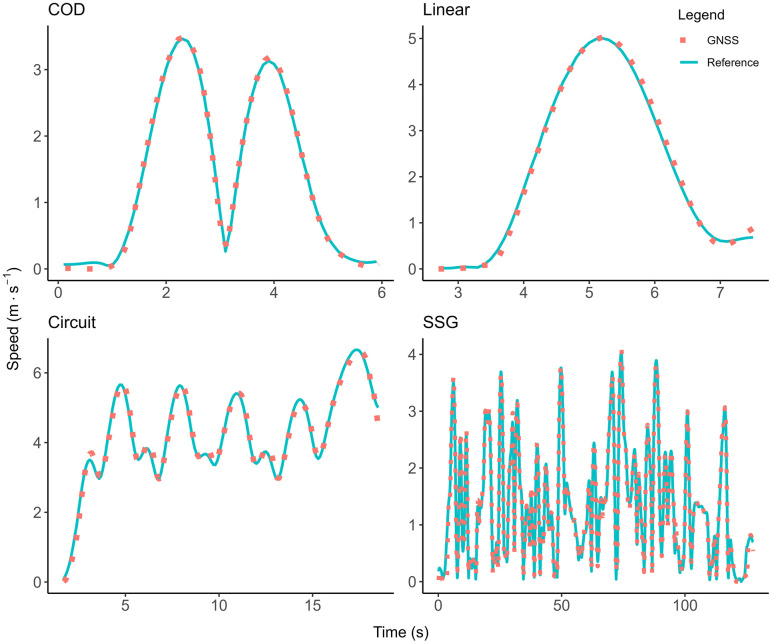
Median mean difference error speed traces. Accuracy of speed data across movement trials with a median mean difference error. The speed traces of the global navigation satellite data (GNSS – red dotted line) are plotted on top of the motion analysis reference speed data (blue line) to show alignment of the data.

**Fig 8:**
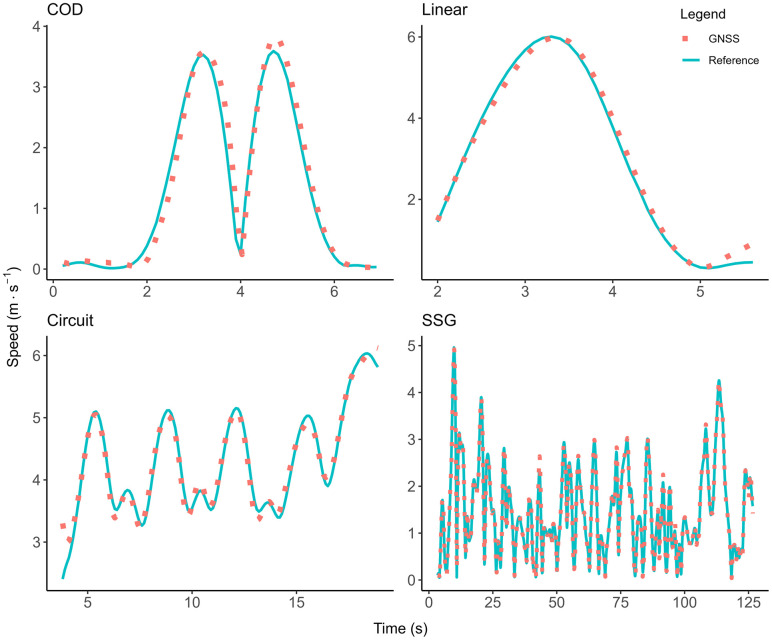
The 95^th^ percentile mean difference error speed traces. Visual representation of the accuracy of speed data across movement trials with a mean difference error in the 95^th^ percentile. The speed traces of the global navigation satellite data (GNSS, red dotted line) are plotted on top of the motion analysis reference speed data (blue line) to show alignment of the data.

The validity results of the GNSS devices compared to the motion analysis criterion for instantaneous data are presented in Table 1, and for accumulated distance data in Table 2. The overall RMSE for instantaneous distance (0.03 ± 0.01), speed (0.14 ± 0.05), and acceleration (0.29 ± 0.14) were minimal and well below the industry standards set by the FIFA quality programme for electronic performance and tracking systems [23]. For example, the RMSE speed threshold set by the FIFA to indicate a well above standard tracking system is ≤ 0.54 m·s^−1^, indicating that a GNSS system with a RMSE value below that threshold is considered well-above standard. The GNSS system tested for this research had a RMSE value of 0.14 ± 0.05 m·s^−1^ which is lower than the FIFA set threshold and thus rated well above standard. There was no overall bias for instantaneous distance and speed, and the bias for acceleration (−0.016) was minimal. The differences between all instantaneous speed and acceleration measurements compared to the motion analysis criterion data are shown in Figs 5 and 6. A visual representation of the accuracy of speed data across movement trials with median mean differences and 95^th^ percentile mean difference are shown in Figs 7 and 8.

**Table 1:** Validity results of instantaneous distance (m), speed (m·s^−1^) and acceleration (m·s^−2^) split by movement trial.

	Instantaneous distance (m)		Instantaneous speed (m·s^−1^)		Instantaneous acceleration (m·s^−2^)	
Movement trial	RMSE (mean±SD)	Mean Bias (90 CI)	RMSE (mean±SD)	Mean Bias (90 CI)	RMSE (mean±SD)	Mean Bias (90 CI)
10 m linear	0.03 ± 0.01	0.00 (0.00, 0.00)	0.15 ± 0.05	0.01 (–0.01, 0.02)	0.36 ± 0.19	−0.01 (−0.07, 0.05)
20 m linear	0.03 ± 0.01	0.00 (−0.01, 0.00)	0.17 ± 0.05	0.00 (–0.02, 0.01)	0.37 ± 0.20	−0.02 (−0.05, 0.01)
COD 180	0.03 ± 0.00	0.00 (0.00, 0.00)	0.13 ± 0.02	0.01 (0.00, 0.02)	0.29 ± 0.05	0.00 (0.00, 0.01)
COD 45	0.03 ± 0.01	0.00 (0.00, 0.00)	0.11 ± 0.03	0.00 (–0.01, 0.01)	0.22 ± 0.06	0.00 (−0.01, 0.01)
COD 90	0.03 ± 0.01	0.00 (0.00, 0.00)	0.13 ± 0.05	−0.02 (–0.04, 0.00)	0.23 ± 0.10	−0.02 (−0.04, −0.01)
Circuit	0.03 ± 0.01	−0.01 (−0.01, 0.00)	0.17 ± 0.04	−0.01 (–0.02, 0.00)	0.39 ± 0.10	−0.07 (−0.08, −0.06)
SSG	0.02 ± 0.00	0.00 (0.00, 0.00)	0.09 ± 0.01	−0.01 (–0.01, 0.00)	0.19 ± 0.02	0.00 (0.00, 0.00)
All	0.03 ± 0.01	0.00 (0.00, 0.00)	0.14 ± 0.05	0.00 (0.00, 0.00)	0.29 ± 0.14	−0.016 (−0.026, −0.005)

RMSE = root mean square error, CI: 90% Confidence Interval, SD = standard deviation.

**Table 2:** Validity results of accumulated distance (m) split by movement trial.

Accumulated distance (m)					
Movement trial	GNSS (mean ± SD)	Criterion (mean ± SD)	RMSE	Mean Bias (m)	Mean Bias %
Linear 10m	10.54 ± 0.48	10.69 ± 0.58	0.26	−0.15	−1.37
Linear 20m	20.13 ± 0.53	20.36 ± 0.60	0.33	−0.23	−1.16
COD 180	9.29 ± 0.45	9.48 ± 0.48	0.29	−0.19	−2.09
COD 45	12.18 ± 0.38	12.37 ± 0.36	0.26	−0.18	−1.52
COD 90	10.12 ± 0.57	10.23 ± 0.49	0.30	−0.11	−1.18
Circuit	69.28 ± 4.06	70.12 ± 3.98	0.94	−0.84	−1.22
SSG	190.66 ± 12.92	193.39 ± 13.49	3.06	−2.73	−1.42

Criterion = 3D motion analysis criterion reference measure, global navigation satellite system (GNSS), RMSE = root mean square error.

**Fig 9:**
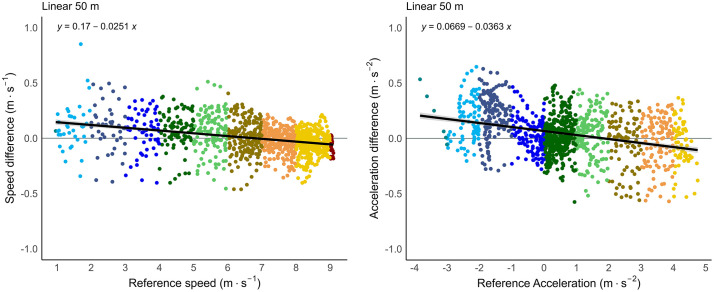
Difference in speed and acceleration of 50 m sprint trials. Instantaneous speed (left) and acceleration (right) error (Criterion – GNSS) of the GNSS during the maximal 50 m sprint trials.

The validity results of the GNSS devices compared to the radar for the 50 m linear run are presented in Table 3. The 50 m linear run had comparable results to the motion analysis trials, with minimal RMSE for instantaneous speed (0.14 ± 0.15) and acceleration (0.22 ± 0.22). The linear trials, together with the circuit trials, displayed larger RMSE values on average compared to the change-of-direction trials, although not significantly different. The difference between all instantaneous speed and acceleration measurements compared to the radar criterion data during the 50 m linear trials are shown in Fig 9.

**Table 3:** Validity results of the 50 m linear sprint trials.

50 m linear sprint trials					
	GNSS (mean±SD)	Criterion (mean±SD)	RMSE	Mean Bias	Mean Bias %
Peak speed (m·s^ − 1^)	8.52 ± 0.44	8.54 ± 0.44	0.05	−0.03	−0.4%
Peak acceleration (m·s^ − 2^)	4.23 ± 0.26	4.25 ± 0.26	0.17	−0.02	−0.5%
Instantaneous speed (m·s^ − 1^)	6.72 ± 1.88	6.72 ± 1.92	0.14 ± 0.15	0.00 (−0.01, 0.01)	
Instantaneous acceleration (m·s^ − 2^)	0.69 ± 1.76	0.65 ± 1.82	0.21 ± 0.20	0.04 (0.03, 0.05)	

Criterion = Radar criterion reference measure, global navigation satellite system (GNSS), RMSE = root mean square error, RMSE is presented as the mean ± standard deviation, Mean Bias is presented with 90% confidence interval.

### Between device reliability

Between device reliability results are presented in Table 4 and demonstrated excellent agreement across all measured variables (ICC ≥ 0.95) with good precision (TE as CV < 3.13%). No significant systematic bias was detected between devices for any variable (p > 0.05).

**Table 4:** Between device reliability results.

	GNSS device #1 (mean±SD)	GNSS device #2 (mean±SD)	Mean Δ (90% CI LOA)	p	ES	ICC (90% CI)	TE as CV% (90% CI)
Total distance (m)	138.9 ± 5.2	139.0 ± 5.2	0.08 (−1.06, 1.22)	0.48	−0.12	0.99 (0.99, 1.00)	0.34 (0.27, 0.41)
Max speed (m·s^−1^)	6.94 ± 0.61	6.95 ± 0.61	0.01 (−0.08, 0.10)	0.30	−0.18	1.00 (0.99, 1.00)	0.52 (0.42, 0.62)
LSR (m)	87.9 ± 9.6	87.8 ± 9.3	−0.09 (−1.85, 1.67)	0.61	0.08	0.99 (0.99, 1.00)	0.83 (0.67, 0.99)
HSR (m)	50.9 ± 6.7	51.1 ± 6.3	0.16 (−1.49, 1.81)	0.34	−0.16	0.99 (0.98, 0.99)	1.35 (1.09, 1.61)
Max acceleration (m·s^−2^)	3.70 ± 0.38	3.67 ± 0.39	−0.03 (−0.23, 0.18)	0.23	0.20	0.95 (0.91, 0.97)	2.39 (1.92, 2.85)
Max deceleration (m·s^−2^)	−3.27 ± 0.38	−3.29 ± 0.38	−0.02 (−0.19, 0.15)	0.07	0.21	0.99 (0.98, 0.99)	−2.22 (−1.79, −2.66)
AccelerationLoad	37.0 ± 2.9	37.1 ± 2.9	0.11 (−1.48, 1.70)	0.48	−0.12	0.95 (0.91, 0.97)	1.78 (1.44, 2.13)
High Metabolic Load Distance (m)	54.7 ± 9.5	54.6 ± 9.7	−0.11 (−4.25, 4.04)	0.92	−0.16	0.97 (0.94, 0.98)	3.13 (2.52, 3.74)

* p < 0.05. Paired T-Test; ES = Cohens d effect size; ICC = interclass correlation coefficient, TE = Typical Error, CV = coefficient of variation, CI = 90% confidence interval; global navigation satellite system (GNSS), LSR = 0–5 m·s^−1^, HSR = > 5 m·s^−1^.

## Discussion

The main findings of this study were that the Catapult Vector S8 GNSS device is valid for measuring instantaneous distance, speed, acceleration and peak speed and acceleration. Furthermore, the measures of total distance, maximal speed, low-speed running (0–5 m·s^−1^), high-speed running (> 5 m·s^−1^), maximal acceleration, maximal deceleration and acceleration load displayed excellent reliability between GNSS devices.

The validity of the Catapult Vector S8 10 Hz GNSS device appeared suitable for measuring instantaneous speed. The validity results are comparable to previous research that used older 10 Hz Catapult devices [1]. The current study had an average RMSE for instantaneous speed of 0.14 compared to 0.15 of the previous similar research. Previous research has shown that instantaneous speed is affected by high changes in speed which mainly occur at lower speeds [1,9,39]. The change in speed from sample to sample can be largest during lower speeds as more force can be applied to the ground compared to higher speeds [40], impacting the validity of the speed data. This is similar to the results of the current study, where data involving lower speeds had reduced accuracy compared to the criterion reference (Fig 5 and Fig 9). However, the error in this study for these data points were low (mean bias instantaneous speed < 0.02 m·s^−1^) and when averaged across all time-series, the errors were negligible (mean bias instantaneous speed = 0.00 m·s^−1^) and were unlikely to affect derived metrics. For example, maximal speed values expected during a soccer game are ~ 9 m·s^−1^ [41], a RMSE of 0.14 is only 1.5% away from the potential maximal speed value. Practitioners can be confident in the validity of the instantaneous speed data derived from the Catapult Vector S8 GNSS device, as error values were close to zero across all conditions.

The instantaneous acceleration of the GNSS across all movement trials was shown to offer suitable validity. Compared to previous research [1], the current study had an average RMSE for instantaneous acceleration of 0.29 compared to 0.39 of the previous similar research. However, the scatterplots in Fig 6 demonstrate a positive trend for instantaneous acceleration where the error increased with acceleration. Both high positive and high negative acceleration values tended to be overestimated, with the latter being underestimated in magnitude. This suggests that the GNSS devices overestimated instantaneous acceleration values during rapid changes of speed. These findings align with observations of previous studies [1,9,39] that highlighted high instantaneous acceleration values are overestimated and likely affected due to the larger point-to-point differences (which for example happens when starting to move from standstill, or performing an abrupt stop from high speed). It is important to note that the acceleration data are derived from the speed data, and the accuracy of the acceleration data is therefore directly related to the accuracy of the speed data. As differentiation amplifies noise, the error in acceleration data (RMSE 0.29 in this study) is also expected to be greater than the speed data (RMSE 0.14 in this study). The trend seen in the plots reinforces that errors in instantaneous acceleration data are linked to both the underlying speed data and the nature of the movement. Despite this, the overall mean error remains low; practitioners and researchers can therefore still confidently rely on instantaneous acceleration data and average acceleration metrics, which are valid and representative of performance across linear and change-of-direction movement sequences.

The measurement error between the radar and GNSS data when measuring maximal speed and acceleration was minimal. The accuracy of GNSS devices to measure maximal speed has been linked to the sample rate, where an increase in sample rate (5–20 Hz) was linked to decreasing typical errors (5.1 to 2.3%) [42]. A more recent paper using a 25-Hz GNSS device showed improved validity with typical errors of 0.5% and a mean bias of −0.07 m·s^−1^ for maximal speed [43]. Furthermore, research using the previous Catapult S7 GNSS devices sampling at 10 Hz have shown to achieve comparable accuracy in maximal speed values to the 20–25 Hz devices, with a reported mean bias for peak speed of −0.05 to −0.08 m·s^−1^ [1,44]. The Catapult S7 GNSS devices also showed good accuracy for maximal acceleration, with reported mean bias of −0.25 m·s^−2^ [1]. The results of the current study showed further improvements in validity with a low mean bias for maximal speed of −0.03 m·s^−1^ and maximal acceleration of −0.02 m·s^−2^. The improved validity of the new Catapult S8 GNSS device compared to the previous Catapult S7 GNSS device for peak speed and acceleration could be due to the improved satellite connectivity of the S8 devices. The current study had an average of 57 connected satellites, compared to 15–16 satellites reported in similar previous research using the S7 device [1,44]. In addition, the reliability of the maximal speed and acceleration were excellent (ICC 0.95–1.0, see Table 4), therefore practitioners and researchers can be confident in their measured maximal speed and acceleration for each GNSS device and between GNSS devices.

The accuracy of the GNSS in measuring accumulated distance showed a small underestimation across all movement trials (mean bias −0.11 to −2.73 m; −1.16 to −2.09%). The RMSE values remained low across trials, ranging from 0.26 m for 10 m linear trials to 3.06 m in the modified SSG, indicating high measurement precision. The modified SSG, despite being the most complex and dynamic trial, still had a low RMSE of 3.06 m compared to similar research [21] who reported a RMSE of 4.90 m for a comparable GNSS device and total distance trial (193 m vs 224 m). Furthermore, the RMSE increased for trials with longer (modified SSG) compared to shorter (COD 45°) distances (see Table 2), but the relative measurement error stayed consistent across trials (mean bias %). Thus, although the error increased as total distance increased (as seen in longer trials like the modified SSG), the degree to which the GNSS underestimated distance remained predictable and consistent. To put the error in context, a professional soccer player has an average total match distance of 11.4 ± 1.0 km [45]. An underestimation of −1.42% would correspond to a difference in total distance of 0.16 km, which is 0.16 standard deviations (SD: 1.0 km) and is considered a small and negligible difference [46]. Overall, the GNSS device provided improved accuracy for accumulated distance, where an average underestimation of −1.42% is expected. The underestimation of distance could be due to the ability of a motion analysis system to detect microscopic movements, which do not result in a noticeable change of position of the participant. These microscopic movements, which may occur as a result of subtle body sway, can only be measured using highly sensitive devices such as a motion analysis system [21]. However, GNSS devices are not designed to measure athlete movements at this level of precision and the results of the current study may therefore underestimate distance.

The between-device reliability showed excellent agreement across all measured variables (ICC ≥ 0.9) with good precision (TE as CV < 5%) see Table 4. The results also showed greater between-device reliability than previous research for measures of total distance, maximal speed, maximal acceleration and deceleration, where the current study had ICC values of 0.95–1.0 compared to 0.2–0.99 in the previous research [44,47]. A custom vest was used for the reliability data collection in the current study, which could result in better reliability results. The custom vest ensured that each GNSS device was positioned in its own pouch, specifically designed for the GNSS device. The use of a custom vest has advantages over using a standard vest, where GNSS devices need to be placed on top of one another. Placing GNSS devices on top of one another [47] can obstruct the GNSS antenna, potentially degrading the data quality. Similarly, using a sled for reliability studies [11,27] has its disadvantages, as the GNSS devices are positioned close to the ground and not worn on the human body. This leads to movement patterns that differ from those experienced when the device is worn on the upper back of an athlete, between the shoulders, where they are typically worn. The high ICC and low TE values observed in this study suggest that the Catapult Vector S8 GNSS device is reliable, and can confidently be used to compare results of different athletes across training sessions and games to guide informed decision-making.

Previous research has shown that different processing practices applied to GNSS data can heavily influence and affect data [27–31]. The results of this study might be superior to comparable previous studies as the manufacturer-specific processing practices used on the criterion data were the same as those used on the GNSS data. This approach ensured that any differences present in the data were not due to the processing practices used, but due to differences in the collected raw data. As this study used the manufacturer-specific processing practises, details of the exact processing could not be disclosed due to intellectual property restrictions. We acknowledge that this limits full reproducibility of the analysis.

A limitation of this study was that the data collection was performed in optimal conditions on an open field with unobstructed line of sight to satellites and no spectators (uncrowded). Although this is applicable to most training sessions for elite team-sports, games usually take place in stadia with spectators, high stands and obstructed line of sight to satellites, potentially leading to less connected satellites and decreased data accuracy. Future research should conduct similar research in stadia and where possible include spectators to simulate real-world scenarios. This will improve the ecological validity of the testing by providing less optimal, real-world testing conditions. The area of testing was not a full-sized team-based sports pitch, which would have allowed for validation testing during game play. However, to the best of the authors knowledge, there is currently no criterion measure (gold standard) that allows full-sized pitch validation testing.

## Supporting information

S1 Dataset Motion analysis database. (XLSX)

S2 Dataset Radar database. (XLSX)
